# Long-term fear of cancer recurrence in patients treated endoscopically for early Barrett’s neoplasia

**DOI:** 10.1093/dote/doac083

**Published:** 2022-12-02

**Authors:** Wilda D Rosmolen, Roos E Pouw, Mark I van Berge Henegouwen, Jacques J Bergman, Mirjam A Sprangers, Pythia T Nieuwkerk

**Affiliations:** Department of Gastroenterology and Hepatology, Amsterdam UMC, University of Amsterdam, Amsterdam, The Netherlands; Department of Gastroenterology and Hepatology, Amsterdam UMC, University of Amsterdam, Amsterdam, The Netherlands; Department of Gastroenterology and Hepatology, Amsterdam UMC, University of Amsterdam, Amsterdam, The Netherlands; Department of Surgery, Amsterdam UMC, University of Amsterdam, Amsterdam, The Netherlands; Department of Medical Psychology, Amsterdam UMC, University of Amsterdam, Amsterdam, The Netherlands; Department of Medical Psychology, Amsterdam UMC, University of Amsterdam, Amsterdam, The Netherlands

**Keywords:** early Barrett’s neoplasia, endoscopic treatment, fear of cancer recurrence

## Abstract

Previous studies on fear of cancer recurrence after endoscopic treatment for early Barrett’s neoplasia focused on fear during a relatively short period after the intervention. The aim of this study was to explore whether fear of cancer (recurrence) persists during long-term follow-up in patients treated endoscopically for Barrett’s neoplasia compared to patients treated surgically for a more advanced stage of esophageal adenocarcinoma. Participants previously participated in a prospective longitudinal study investigating quality of life and fear of cancer recurrence and were treated endoscopically for early Barrett’s neoplasia (high-grade dysplasia—T1sm1N0M0) or surgically for a more advanced esophageal adenocarcinoma (T1N0M0–T3N1M0). For the present study, participants were again invited to complete a set of questionnaires including the fear of cancer recurrence scale (FORS), worry for cancer scale (WOCS), and the anxiety subscale of the Hospital Anxiety and Depression Scale (HADS Anxiety). Thirty-nine patients were eligible in the endoscopy group and 28 in the surgical group. The median time between the baseline measurement (original study) and the long-term follow-up assessment was 4 years (interquartile range 3–5 years). Fear and worry for cancer recurrence and general anxiety diminished over time in both treatment groups. However, at long-term follow-up, endoscopically treated patients had significantly higher levels of worry for cancer and general anxiety than surgically treated patients. Fear of cancer recurrence did not significantly differ between endoscopically and surgically treated patients. We found that worry and fear of cancer recurrence and general anxiety in endoscopically treated patients declined over time, but not as much as in surgically treated patients.

## INTRODUCTION

Endoscopic treatment is a minimally invasive and organ-preserving alternative to surgery for curation of early neoplasia in Barrett’s esophagus (i.e., high-grade dysplasia and esophageal adenocarcinoma).^1^^,^^2^ Given its excellent efficacy and long-term durability, endoscopic treatment is nowadays the treatment of choice for early Barrett’s neoplasia.^1^^,^^3^^,^^4^^,^^5^ In a prior prospective study, we investigated quality of life (QOL) and fear of cancer recurrence at baseline, 2, and 6 months after treatment for Barrett’s neoplasia. We compared outcomes in endoscopically treated patients with high-grade dysplasia and stage T1a/low risk T1b esophageal adenocarcinoma with three reference groups: patients treated surgically for early Barrett’s cancer, patients treated surgically for advanced Barrett’s cancer, and patients with non-dysplastic Barrett’s esophagus undergoing surveillance.^6^ We found that the reported QOL of patients treated endoscopically was comparable to that of patients with non-dysplastic Barrett’s esophagus. Patients in the endoscopic treatment group had significantly better scores on almost all functional scales of the SF-36 and the EORTC-QLQ-C30 compared to the two surgical groups.^6^ An unexpected finding of our prior study was that patients treated endoscopically for early Barrett’s neoplasia did not report a lower level of worry for cancer (recurrence) as patients treated surgically for more advanced esophageal cancer.^6^^,^^7^ This worry for cancer (recurrence) was reported despite excellent long-term results of endoscopic treatment with more than 95% 5-year survival rates and low recurrence rates.^2^^,^^4^ Studies among patients who were treated for other types of cancer, e.g., early breast cancer, thyroid cancer, ovarian cancer, gastric cancer or prostate cancer, also found substantial fear of cancer recurrence.^4^^,^^8^^,^^9^ There are no conclusive data on fear of cancer recurrence in relation to cancer stage or follow-up time.^10^^,^^11^ There is some evidence that patients who undergo follow-up procedures after cancer treatment report more fear of cancer recurrence.^9^^,^^12^^,^^13^ The question arises whether fear of cancer recurrence persists over time in patients with early Barrett’s neoplasia treated endoscopically. Therefore, we used the baseline (before treatment) data of our previous prospective study that explored fear of cancer recurrence in patients treated endoscopically for early Barrett’s neoplasia or surgically for more advanced stage of esophageal adenocarcinoma and compared these data with new long-term follow-up data.

## PATIENTS AND METHODS

### Patients

Patients from the previously reported prospective study on QOL and fear of cancer recurrence were approached for participation in this long-term follow-up study.^6^ Patients treated endoscopically for early Barrett’s neoplasia (high-grade dysplasia—T1sm1N0M0) were compared with patients treated surgically for more advanced (T1N0M0–T3N1N0M0) esophageal adenocarcinoma.

### Ethical considerations

The Amsterdam UMC research board exempted the study from further ethical review since the Medical Research Involving Human Subjects Act (WMO) does not apply.

### Demographic and clinical data

Demographic data were assessed at baseline by self-report. Clinical data, including co-morbidity, type of treatment, the duration of the treatment, histology results, complications, recurrence during follow-up and additional treatment, were extracted from medical charts and the web-based hospital information system.

### Procedure

In the previous study, patient enrollment took place from January 2006 to March 2011. Fear of cancer recurrence data were analyzed and results were published.^6^ In the previous study, patients treated for early esophageal adenocarcinoma (≤T2N0M0) and patients treated surgically for advanced esophageal adenocarcinoma (≥T1N1M0) were kept separate as two distinct groups. However, at the time of the long-term follow-up assessment, a substantial number of patients in the advanced group had died of esophageal cancer, leaving the advanced group with eight patients. Because of the small group size, we combined the early and advanced surgical group into one (surgical) group. The long-term follow-up study was initiated in April 2012. The same patient-reported outcome measures were mailed to participants known to be alive in April 2012. A reminder was sent after 2 weeks.

### Questionnaires

*Fear of cancer (recurrence)* was measured with the Worry for Cancer Scale (WOCS) and the Fear of Recurrence Scale (FORS). The WOCS is a four-item questionnaire and specifically targets fear of esophageal cancer. An example of one of the questions is: ‘To what extent does worry about the esophageal illness spill over or intrude on your other thoughts and activities?’ The response options range from ‘not at all’ (0) to ‘a great deal’.^10^ The maximum score is 40, with a higher score indicating more worry.^14^ The FORS consists of 22 items, with the response options strongly agree, agree, neutral, disagree, and strongly disagree. The score ranges from 22 to 110 with a higher score indicating more fear of recurrence. This questionnaire measures fear of recurrence in general and is also suitable to measure fear of recurrence in other diseases.^15^^,^^16^ The items of the WOCS and the FORS can be viewed in Supplement 1.

The general level of anxiety was assessed with the 7-item anxiety subscale of the Hospital Anxiety and Depression Scale (HADS Anxiety) to assess fear in general and not specifically related to Barrett’s/esophageal cancer. The response options range from 0 to 3 and have different labels across items and range. The maximum score is 21. Scores between 0 and 7 suggest no problems of clinical relevance. A score between 8 and 10 justifies further psychological evaluation. A score of 11 or higher is indicative of clinical levels of general anxiety.^17^^,^^18^

**Fig. 1:**
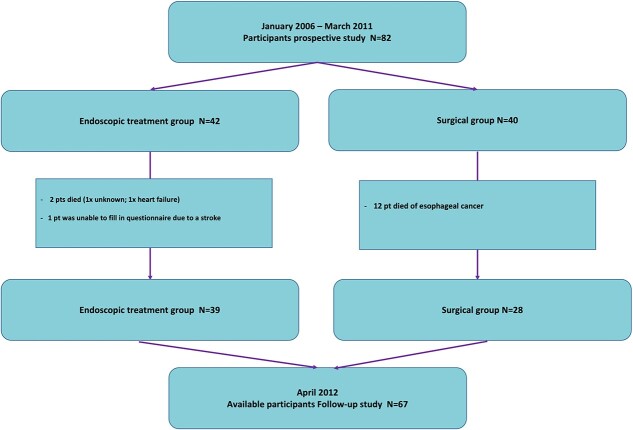
Flowchart of patient inclusion.

### Statistical analyses

SPSS (IBM version 26.0) was used for the statistical analyses. Baseline demographic characteristics and clinical data of the patients included in this long-term follow-up study were compared using independent *t*-tests for continuous data and the chi-square tests for categorical data. We investigated changes in worry of cancer, fear of cancer recurrence, and anxiety in general from baseline to the long-term follow-up measurement within each treatment group using paired samples *t*-tests. We compared long-term worry of cancer, fear of cancer recurrence, and anxiety in general between the group of patients who had been treated endoscopically for early Barrett’s neoplasia versus patients who had been treated surgically for more advanced esophageal adenocarcinoma using analysis of variance with the baseline measurement of the respective scales included as covariate (ANCOVA). Two-sided *P*-values of <0.05 were considered to be statistically significant. To indicate clinical relevance, effect size was calculated for the change in WOCS, FORS, and HADS Anxiety  scores over time by dividing the mean change from by the standard deviation at baseline. The effect size for the difference between the two groups at the long-term follow-up measurement was calculated by the mean difference between the two groups, adjusted for the baseline measurement, age, and gender, divided by the pooled standard deviation at baseline.

## RESULTS

At baseline, 82 participants (Endoscopy group *n* = 42; surgical group *n* = 40) entered the study. At time of the long-term follow-up assessment, 67 participants (endoscopy group *n* = 39; surgical *n* = 28) were eligible (Figure 1). Only one of the eligible participants in the surgical group did not complete the follow-up questionnaire, therefore the response rate of the follow-up questionnaire was 98.5%. The mean time between the baseline assessment of the original prospective study and completion of the long-term follow-up assessment was 4 years (interquartile range 3–5 years). The following demographic and clinical characteristics: age, gender, and co-morbidity from the participants of follow-up assessment did not significantly differ between the two groups at baseline (Table 1).

**Table 1:** Demographic and clinical data

	Endoscopic group *N* = 39	Surgical group *N* = 27	*P*-value
Demographic data at baseline			
Gender (male %)	87%	89%	0.576
Age (mean, SD)	62 (10.2)	64 (7.8)	0.388
Clinical data at baseline			
Co-morbidity (%) – None – One – Two or more	41% 31% 28%	44% 26% 30%	0.922
Clinical data during treatment			
Treatment-related complications	3%	59%	**0.000**
Histology (%) – Low-grade dysplasia – High-grade dysplasia—T1sm1N0M0 – T1sm2N0M0–T2N0M0 – T1N1M0–T4N1M0	1% 99%	44% 30% 26%	**0.000**

*P* values <0.05 were considered to be significant. SD, standard deviation.

Patients in the endoscopy group had annual surveillance endoscopies after their primary endoscopic treatment. During follow-up, recurrence of mucosal adenocarcinoma was found in two of these patients. One patient was subsequently successfully treated endoscopically. In the other patient, focal low- and high-grade dysplasia was found, and the mutual decision was made not to perform additional treatment, but to keep the patient under strict endoscopic follow-up. Surgical patients were clinically followed; no standard medical examinations were performed. Twelve patients in the surgical group died of esophageal cancer recurrence (Figure 1).

### Fear of cancer (recurrence) and general anxiety over time

Compared to baseline, the mean scores of fear of cancer (recurrence)—the WOCS and FORS—and general anxiety—HADS Anxiety scale—declined significantly over time in both endoscopically treated patients and surgically treated patients (*P*-values in both the endoscopy group and surgical group <0.05 on the WOCS, FORS, and HADS Anxiety). The calculated effect sizes were moderate to large (0.35–1.55) (Table 2).

**Table 2:** WOCS, FORS, and HADS Anxiety scores at baseline and long-term follow-up

	Endoscopy group (*n* = 39				Surgical group (*n* = 27)			
	Baseline	Long-term follow-up	*P*-value	Effect size	Baseline	Long-term follow-up	*P*-value	Effect size
WOCS	14.9	9.33	0.000	0.76	14.9	6.2	0.000	1.55
FORS	67.3	57.5	0.000	0.60	75.1	60.5	0.000	1.69
HADS Anxiety	5.0	3.6	0.008	0.35	6.1	2.7	0.000	0.99

On the WOCS, scores rate between 0 and 40; on the FORS, scores rate between 22 and 110. A higher score on both WOCS and FORS indicates more fear of cancer (recurrence). On the HADS Anxiety, scores rate between 0 and 21. Scores between 0 and 7 suggest no problems of clinical relevance. *P* values <0.05 were considered to be significant. FORS, Fear of Recurrence Scale; HADS Anxiety, anxiety subscale of the Hospital Anxiety and Depression Scale; WOCS, Worry for Cancer Scale.

### Comparison between endoscopically and surgically treated patients at long-term follow-up

At long-term follow-up, the endoscopic treatment group had a significantly higher score on the WOCS (mean scores: 9.5 vs. 6.2; *P* 0.036) and the HADS Anxiety scale (mean scores: 3.5 vs. 1.8 *P* 0.011) compared to the combined surgical group. However, both groups had a mean scores <7 on the HADS Anxiety scale, indicating that clinically relevant levels of general anxiety or depression were not present. At long-term follow-up, no significant differences were found in the FORS between the endoscopic treatment group and the combined surgical groups (mean scores: 59.7 0 vs. 56.6; *P* 0.308). Calculated effect sizes for the WOCS, HADS Anxiety, and FORS were, respectively, 0.49, 0.34, and 0.23 (Table 3).

**Table 3:** Mean scores of the long-term follow-up, adjusted for baseline, age, and gender

	Endoscopy group *N* = 39	Surgical group *N* = 27	Endoscopy versus surgical *P*-value	Effect size
WOCS	9.5	6.2	0.036	0.49
FORS	59.7	56.6	0.308	0.23
HADS Anxiety	3.5	1.8	0.011	0.34

On the WOCS scores, rate between 0 and 40; on the FORS scores, rate between 22 and 110. A higher score on both WOCS and FORS indicates more fear of cancer (recurrence). On the HADS Anxiety, scores rate between 0 and 21. Scores between 0 and 7 suggest no problems of clinical relevance. *P* values <0.05 were considered to be significant. FORS, Fear of Recurrence Scale; HADS Anxiety, the anxiety subscale of the Hospital Anxiety and Depression Scale; WOCS, Worry for Cancer Scale.

## DISCUSSION

In a period of 3–5 years after surgical or endoscopic treatment for esophageal adenocarcinoma, fear and worry for cancer recurrence and general anxiety diminished compared to baseline measurements. Endoscopically treated patients, however, reported about the same level of fear of cancer recurrence and significantly higher levels of cancer worry and general anxiety compared to surgically treated patients.

Fear of cancer recurrence is common in patients surviving cancer. It is estimated that 70% of all cancer survivors irrespective of stage experience fear of cancer recurrence. It appears that fear of cancer recurrence is not specifically related to a specific type of cancer.^8^^,^^9^ Also, our study adds to the number of studies that did not find a clear relationship between fear of cancer recurrence and cancer stage.^6^^,^^7^^,^^10^^,^^12^ However, two other studies have shown that stage I breast cancer patients report less fear of recurrence compared to stage II breast cancer patients.^12^ Mixed results on fear of cancer recurrence were also found when patients undergoing organ sparing surgery were compared with those undergoing complete organ resection. The results, however, were mostly based on studies with breast cancer survivors.^12^ In the present study, we found that patients in both the endoscopic and the surgical groups reported higher levels of fear of cancer (recurrence) and general anxiety at baseline. Furthermore, fear of cancer (recurrence) and general anxiety did not significantly differ in both groups at baseline. Since the cancer was still present at baseline, this outcome is to be expected. The present long-term follow-up results show that general anxiety and worry for cancer declined, albeit to a lesser extent in patients treated endoscopically compared to surgically treated patients. Prior research with a shorter follow-up period showed similar results.^6^^,^^7^

The question remains why early esophageal cancer combined with minimally invasive treatment leads to more worry and anxiety and a similar level of fear for cancer (recurrence) compared to a more advanced stage of esophageal cancer combined with invasive treatment. There is some evidence that patients who undergo follow-up procedures after cancer treatment report more fear of cancer recurrence.^9^^,^^12^^,^^13^ This might explain the worry, anxiety, and fear of cancer recurrence in patients treated endoscopically for early Barrett’s neoplasia, as these patients are subjected to a regular endoscopic follow-up protocol with yearly endoscopic inspection. Conversely, surgically treated patients undergo clinically follow-up, with further investigations only in case of symptoms.^13^ Also insufficient information about recurrence rates and reflux and dyspeptic-related symptoms which patients may experience presumably lead to fear of cancer recurrence. Patient education may be a sufficient tool to provide patients treated endoscopically for early esophageal adenocarcinoma with information. However, literature about patient education reducing fear of cancer recurrence is limited, and the results that are available suggest that the effect of patient education on reducing fear of cancer recurrence is also limited.^19^

Strengths of the present study include the long-term follow-up period, the use of a well-documented group of patients, and the use of a comparison group. Furthermore, standardized and validated questionnaires were used and the response rate of the patients to the long-term follow-up questionnaire was 99%. A possible explanation for the high response rate could be the fact that the care for patients with early esophageal adenocarcinoma is provided by highly involved caregivers, which may lead to the willingness to participate.

Also some limitations need to be addressed. First, the sample size of the study was small to begin with and 12 patients in the original advanced surgical group deceased, reducing the control group to 64% of the original cohort. This is why the patients from the early surgical group and the advanced surgical group were combined into one surgical group. Furthermore, the target population of this study is the patients that are treated endoscopically. Second, whereas the patients in the endoscopy group reported a significantly higher score on the WOCS, compared to the surgically group, we are uncertain whether this difference was also clinically relevant as clinically relevant cut-off scores of the WOCS are lacking. However, the moderate-to-large effect sizes are indicative of clinically relevant differences between the two groups. Third, the long-term follow-up data were collected 10 years ago. However, although the changes in endoscopic resection techniques, with a shift towards endoscopic submucosal dissection, the algorithm for endoscopic treatment and follow-up surveillance endoscopies did not change in the last decade. The treatment of patients with early Barrett’s neoplasia is performed by the same medical team over more than a decade, consisting of two endoscopists and two nurse practitioners. Endoscopic resection is still followed by radio frequency ablation as in this study cohort. Furthermore, we do not think that the used resection technique does influence the study outcome. We therefore believe our data are still relevant.^1^^,^^20^ Fourth, except for recurrence rates and the following (endoscopic) treatments, other clinical data were not taken into account. Finally, the original study focused on QOL and fear of cancer recurrence. We did not include QOL in this study because the results of the original study showed that QOL was not influenced by endoscopic treatment 6 months after treatment. We anticipated that QOL would not be negatively influenced by endoscopic treatment after 4 years. Furthermore, we would have a lot of outcome measures compared to number of patients participating the study.

The prospects of patients with early esophageal adenocarcinoma are excellent.^1–4^ However, as demonstrated in this study, worry, fear of cancer recurrence, and general anxiety in endoscopically treated patients are still a relevant finding, even years after successful treatment. As clinicians we have to realize that fear and worry for cancer recurrence are real and the impact of cancer diagnosis may be profound. We need to explore and address patients’ fears and concerns actively. Future studies should explore the reasons, why patients with early esophageal cancer may experience worry, fear of cancer recurrence, and anxiety.^6–10^^,^^12^

## Conflicts of interest

Jacques J. Bergman has received unrestricted grants for research support from Cernostics, Olympus Endoscopy, Aqua Medical, and Medtronic; financial support for training programs from Medtronic and Pentax; consultancy fee from Olympus, Fractyl, Endogenex, Digma, Aqua Medical; study sponsoring from Fractyl Laboratories, Endogenex, Digma, CDx Diagnostics, and Lucid. Roos E. Pouw has received speaker’s fee from Medtronic for participation in courses and consultancy fee for MicroTech. Mark I. van Berge Henegouwen has received a grant for research support from Stryker, Consultancy fee from Johnson and Johnson, Alesi Surgical, Mylan, BBraun, and Medtronic. All other authors disclosed no financial relationships relevant to this publication. The data underlying this article cannot be shared publicly due to the privacy of individuals that participated in the study. The data will be shared on reasonable request to the corresponding author. This research received no specific grant from any funding agency in the public, commercial, or not-for-profit sectors.
